# CTLA-4 expression in the non-small cell lung cancer patient tumor microenvironment: diverging prognostic impact in primary tumors and lymph node metastases

**DOI:** 10.1007/s00262-017-2039-2

**Published:** 2017-07-13

**Authors:** Erna-Elise Paulsen, Thomas K. Kilvaer, Mehrdad Rakaee, Elin Richardsen, Sigurd M. Hald, Sigve Andersen, Lill-Tove Busund, Roy M. Bremnes, Tom Donnem

**Affiliations:** 1 Department of Oncology, University Hospital of North Norway, Mailbox 13, 9038 Tromso, Norway; 2 Department of Clinical Medicine, UiT The Arctic University of Norway, Mailbox 6050, Langnes, 9037 Tromso, Norway; 3 Department of Clinical Pathology, University Hospital of North Norway, Mailbox 46, 9038 Tromso, Norway; 4 Department of Medical Biology, UiT The Arctic University of Norway, Mailbox 6050, Langnes, 9037 Tromso, Norway; 5 Translational Cancer Research Group, Department of Clinical Medicine, UiT The Arctic University of Norway, Mailbox 6050, Langnes, 9038 Tromso, Norway

**Keywords:** CTLA-4, Prognostic, Non-small cell lung cancer, Immune checkpoints, Immunoscore

## Abstract

The immune checkpoint receptor CTLA-4 plays a crucial part in negatively regulating T cell activation and maintaining self-tolerance. It is frequently overexpressed in a variety of malignancies, yet its prognostic impact in non-small cell lung cancer (NSCLC) remains unclear. We constructed tissue microarrays from tumor tissue samples and evaluated the immunohistochemical expression of CTLA-4 in 536 patients with primary resected stage I–IIIA NSCLC. Expression of CTLA-4 was analyzed in tumor and stromal primary tumor tissue and in locoregional metastatic lymph nodes. CTLA-4 expression in neither tumor epithelial cells (T-CTLA-4) nor stromal cells (S-CTLA-4) of primary tumors was significantly associated with disease-specific survival (DSS) in all patients. However, high S-CTLA-4 expression independently predicted significantly improved DSS in the squamous cell carcinoma subgroup (HR 0.62, 95% CI 0.41–0.93, *P* = 0.021). In contrast, there was an independent negative prognostic impact of T-CTLA-4 expression in metastatic lymph nodes (HR 1.65, 95% CI 1.03–2.65, *P* = 0.039). Our results indicate that the expression of CTLA-4 has diverging prognostic impacts in metastatic NSCLC lymph nodes versus primary tumors. The presented results highlight important differences in the tumor microenvironments of primary and metastatic NSCLC tissues, and have potential to guide treatment and clinical sampling strategies.

**Electronic supplementary material:**

The online version of this article (doi:10.1007/s00262-017-2039-2) contains supplementary material, which is available to authorized users.

## Introduction

Non-small cell lung cancer (NSCLC) mortality is high and there is a strong need for novel prognostic biomarkers to improve prediction of patient outcomes, to aid clinical decision-making and increase survival [1, 2]. Following the recent impressive results observed targeting the immune system in cancer therapy, biomarker research focus has turned to the tumor microenvironment. Different subsets of tumor infiltrating immune cells interact with malignant cells in a complex and dynamic ecosystem, mediating immune surveillance and destruction of cancer cells as well as pro-tumoral inflammation [3, 4]. In fact, extensive research supports that the immune contexture has an impact on cancer patient outcomes [5]. Recently, quantification of the in situ immune infiltrate was found to supplement the prognostic accuracy of the TNM classification in breast cancer, colorectal cancer and NSCLC, the two latter applying an “Immunoscore” method [6–8].

CTLA-4 (Cytotoxic T-lymphocyte-associated antigen-4, CD152) is the receptor of an immune checkpoint pathway that plays a crucial role in the regulation of T cell activation and preservation of self-tolerance. Hence, its expression in the tumor microenvironment constitutes a potential prognostic and predictive biomarker in NSCLC patients [9].

T cells are activated in secondary lymphoid organs, when the T cell antigen receptor recognizes antigen-MHC complexes on antigen-presenting cells (APCs). A costimulatory signal is elicited by the engagement of CD28 on T cells with B7 ligands (CD80 and CD86) on APCs [10]. However, upon T cell activation, the CD28 homologue CTLA-4 is translocated from intracellular storage to the plasma membrane of T cells, competitively binding to B7 ligands on APCs with higher affinity, thereby preventing CD28-mediated T cell activation [9]. While naïve T cells upregulate CTLA-4 only after activation, regulatory T cells constitutively express CTLA-4, and the result of CTLA-4 ligation is mediation of the suppressive function of regulatory T cells and inhibition of conventional T cells, but the exact molecular mechanisms remain to be elucidated [11, 12]. Expression in non-T cell subsets has also been observed, though its role remains uncertain [13–17].

Sustained overexpression of CTLA-4 is often induced in chronic inflammation and cancer, implying that CTLA-4 in the tumor microenvironment may be involved in dysregulation of the immune response in cancer [18, 19]. Moreover, monoclonal antibodies targeting CTLA-4 enhance T cell mediated anti-tumor immunity [20–22]. Yet, studies examining the prognostic impact of CTLA-4 expression in NSCLC tumor tissue are few and inconclusive [23–26]. How CTLA-4 expression is distributed in primary tumors (PTs) and metastatic sites, and how this might influence patient outcome, is presently unclear. Hence, to increase the understanding of the natural course of NSCLC, further research on the roles of CTLA-4 expression in NSCLC, which can potentially guide treatment preferences and tumor sampling strategies, is needed.

Hypothesizing that CTLA-4 is a candidate prognostic biomarker for inclusion in a NSCLC Immunoscore, we aimed to explore the prognostic impact of CTLA-4 in tumor epithelial and stromal cells of PTs from 536 resected stage I-IIIA NSCLC patients as well as in 142 matched lymph node metastases (LN+).

## Materials and methods

### Patients and clinical samples

PT tissues from an unselected patient population who underwent radical resection for NSCLC pathologic stage I to IIIA at the University Hospital of North Norway and the Nordland Hospital from 1990 to 2010, were retrospectively collected. In total, 536 patients with complete medical records and adequate paraffin-embedded tissue blocks were eligible, as previously described, including 142 patients with available lymph node specimens out of the 172 patients with N+ disease [27]. This report includes follow-up data as of October 1, 2013. Median follow-up time of survivors was 86 months (range 34–267 months). The Norwegian Data Protection Authority and the Regional Committee for Medical and Health Research Ethics approved the study (Protocol ID: 2011/2503), and the need for patient consent was waived. Reporting of clinicopathological variables, survival data, and biomarker expressions was conducted in accordance with the REMARK guidelines [28].

### Microarray construction

All tissues were histologically reviewed by two pathologists. The most representative areas of viable neoplastic epithelial cells and of tumor stroma in the PT and matched LN+ were carefully selected for the tissue microarrays (TMAs). Cores were not consistently taken from specific tumor areas, such as central tumor or invasive margin. TMAs were assembled using a tissue-arraying instrument (Beecher Instruments, Silver Springs, MD, USA). The detailed methodology has been previously reported [29]. Briefly, we used a 0.6-mm diameter stylet. Four cores were sampled from different areas in the two compartments, two from tumor epithelium and two from tumor stroma.

### Immunohistochemistry

The antibodies evaluated for CTLA-4 expression were mouse monoclonal CD152 (eBioscience, clone: 14D3, Cat#14-1529) and rabbit polyclonal CTLA-4 (Abcam, Cat#ab151773). Antibodies were subject to in-house validation by the manufacturer. In addition, we performed validation by staining multi-organ TMAs as positive and negative tissue controls, and transfectant plasmid cell lysates (See CD152 Antibody Validation). Normal placenta sections served as positive tissue controls, negative tissue controls comprised sections of normal brain tissue. Only the CD152 (clone 14D3) antibody fulfilled the standards for evaluation and was used in this study.

Immunohistochemical analyses were performed on Discovery-Ultra immunostainer (Ventana Medical Systems, Tucson, AZ). Slides were deparaffinized in three 8-min cycles, and heated overnight at 60 °C. For on-board antigen retrieval, slides were incubated with Cell Conditioning Solution 1 (CC1) buffer for 24 min. Endogenous peroxidase was blocked by Discovery inhibitor (Cat#760-4840) for 8 min. The CD152 primary antibody in 1/100 dilution was loaded and slides were incubated for 32 min at 37 °C. Slides were developed using OmniMap anti-mouse HRP (Cat#760-4310) for 20 min, followed by chromogenic detection kit ChromoMap DAB (Cat#760-159). Finally, to visualize the nuclei, all slides were counterstained with Ventana Hematoxylin II reagent (Cat# 790-2208) for 32 min, followed by a Bluing reagent (Cat# 760-2037) for 8 min. Slides were then dehydrated, cleared and mounted as in routine processing. Control staining by (1) omission of the primary antibody and by (2) incubation with a subclass isotype-matched control antibody (Biolegend, Cat#400203), omitting the CD152 primary antibody, was also performed.

### CD152 antibody validation

Cell lysates from CTLA-4 transiently transfected HEK293 cells (HEK293T, Cat#LY417438) and from empty vector transfected cells (HEK293, Cat#LY500001/negative control) were applied from OriGene; they were incubated with 2xSDS Sample Buffer for 10 min at 100 °C. Equal amounts of protein lysates were resolved onto a 4–12% Bis–Tris gel (Cat#NP0322, Life Technologies). The resolved proteins were transferred onto an Odyssey nitrocellulose membrane (#926-31092, LI-COR), and the membrane was subsequently blocked for 1 h at room temperature using the Odyssey blocking buffer (Cat#927-40000, LI-COR). CD152 antibody (clone 14D3) in a 1/100 dilution was applied, and the membrane incubated overnight at 4 °C. Subsequently, goat anti-mouse IRDye 800CW secondary antibody (Cat#926-32210, LI-COR) in 1/10.000 dilution was added, and incubated for 1 h at room temperature. Between antibody incubations, the membrane was washed three times for 5 min each time in tris-buffered saline containing 0.05% Tween 20 (Sigma-Aldrich). Molecular weight markers used were MagicMark XP Western Protein Standard (LC5603, Invitrogen) and SeeBlue Plus2 Pre-stained Standard (#LC5925, Invitrogen). The most prominent bands represent the observed molecular weight (30 kDa) of the detected protein, which corresponds intimately with the predicted weight provided by the manufacturer (24.66 kDa) (supplementary Fig. 1). Rabbit anti-actin (Cat#A2066, Sigma-Aldrich), 1:1000, was used as internal control and the lanes show 42 kDa molecular weight protein load.

### Scoring of IHC

Samples were anonymized and independently scored by two of the authors (TK and EP), under the supervision of an experienced pathologist (ER), who established a semi-quantitative score for each marker. When assessing a given core, the observers were blinded to each other, to clinical variables and to outcome. Tumor epithelial and stromal compartments were scored separately. Strongly staining cells morphologically consistent with macrophages were excluded from scoring, as the staining may represent ingested debris rather than macrophage staining.

**Fig. 1:**
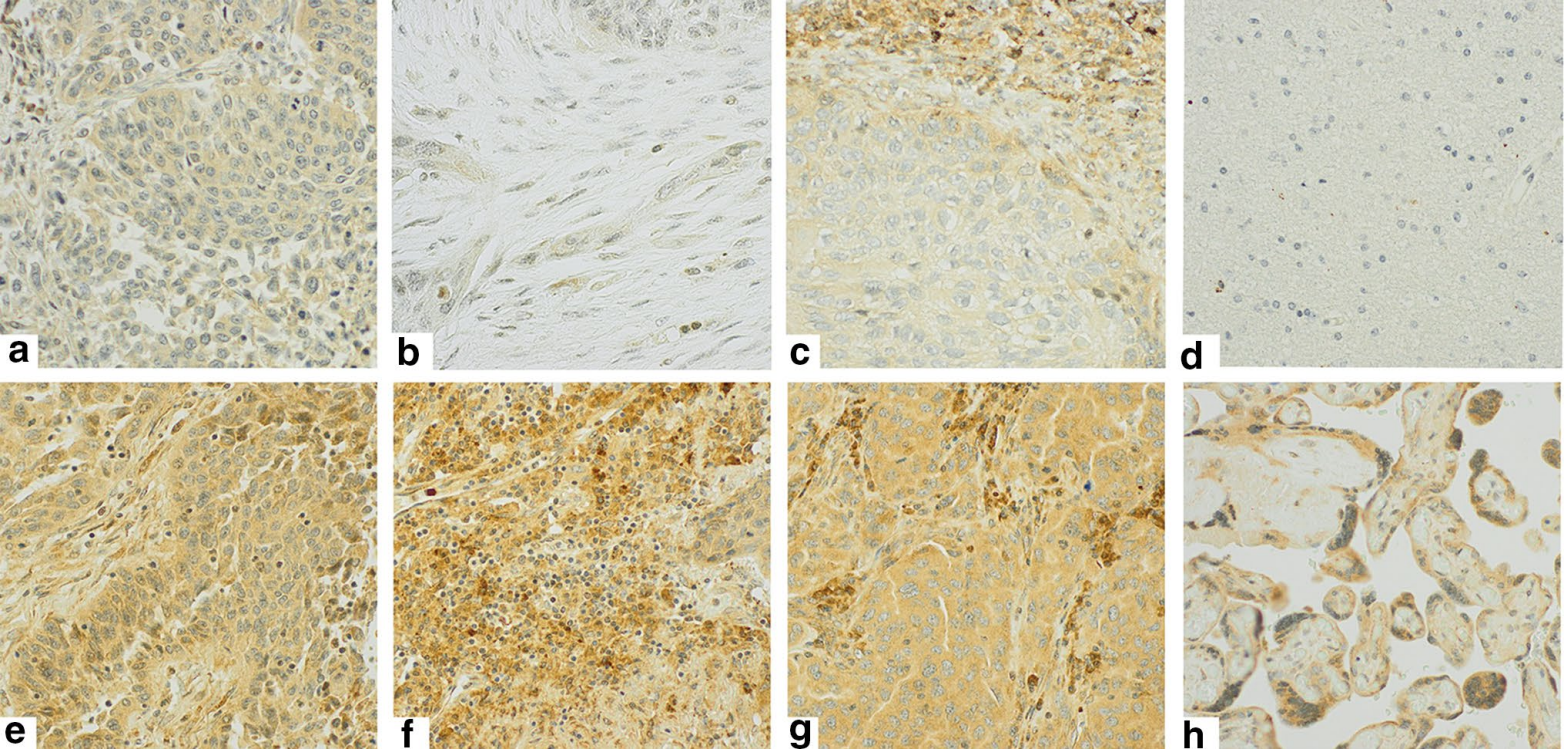
CTLA-4 immunohistochemical analysis in primary tumors and metastatic lymph nodes. Immunohistochemical analysis of non-small cell lung cancer representing low and high scores for tumor cell CTLA-4 expression in PTs (T-CTLA-4: **a**, **e**), stromal expression in PTs (S-CTLA-4: **b**, **f**), tumor cell expression in LN+ (T-CTLA-4: **c**, **g**), negative (**d**, brain) and positive tissue controls (**h**, placenta). Magnification x 400

Because staining of CTLA-4+ cells was relatively homogenous, both within tumor cells in the tumor epithelial compartment and within the different stromal cells in the stromal compartment, the percentage (density) of CTLA-4 positive cells did not add valuable information to the score, and was not included. Intensity of CTLA-4 staining was scored as 0 (no staining), 1 (weak), 2 (moderate), and 3 (strong) in both the tumor epithelial (T-CTLA-4) and stromal (S-CTLA-4) compartments. As CTLA-4 staining was present in both tumor epithelial cells and immune cell subsets infiltrating the tumor epithelium, we were unable to precisely evaluate and score CTLA-4+ intraepithelial immune cells due to overlap of the chromogenic DAB substrate. Identical scoring approaches were used in PTs and LN+. Two cores were sampled from each compartment (tumor epithelial and stromal) and scored by two individuals, hence four or two (if one TMA core was missing) scores were available. Based on the mean value of these scores, the threshold value for dichotomization of patients was determined. The cutoff that resulted in the minimal *P* value with regard to difference in outcome between the two groups was chosen (optimal cutoff). Accordingly, a high score was defined as >2.00 for S-CTLA-4 and >1.25 for T-CTLA-4. Figure 1 illustrates IHC scoring of CTLA-4 in PTs and LN+.

Scoring of other immunological markers previously analyzed by our group: CD3, CD4, CD8, CD45RO, CD20, PD-1 (programmed death 1 receptor), PD-L1 (programmed death ligand 1), has been previously described [27, 30–33].

### Statistical methods

All statistical analyses were performed using the SPSS statistical package (version 22, SPSS, Chicago, IL, USA). The IHC scores from each observer were compared for interobserver reliability by use of a two-way random effects model with absolute agreement definition, yielding an intraclass correlation coefficient (reliability coefficient) and Cohen’s kappa. DSS (disease-specific survival) was defined as the time from surgery to lung cancer death.

The *χ* ^2^ test or Fisher’s exact test was used to examine the association between molecular marker expression and various clinicopathological parameters. Spearman’s rank correlation was used to examine the associations between marker expressions. Univariate analysis of survival according to each immune marker was visualized using the Kaplan–Meier method, and statistically significant differences between survival curves were assessed by the log-rank test. For univariate analyses, unadjusted Cox proportional hazard ratios were calculated. Multivariate analysis was performed using the Cox proportional hazards model, testing the simultaneous influence on survival of all covariates found to be significant in the univariate analyses. The backward conditional method was used for model fitting. Probability for stepwise entry and removal was set at 0.05 and 0.10, respectively. *P* values < 0.05 were considered statistically significant. Correction for multiple testing was not performed, as the study is of an exploratory, hypothesis-generating nature.

## Results

### Patient characteristics

Demographic, clinical and histopathological variables for all 536 patients and their impact on DSS are presented in Table 1. Of the 172 patients with N+ disease, 142 had adequate paraffin-embedded tumor specimens from tumor, and were included in this study (Clinicopathological variables of N+ patients is presented in supplementary Table 1). Median age was 67 (range 28–85) years and 68% of the patients were men. Due to nodal metastasis or non-radical surgical margins, 76 patients (14%) received postoperative radiotherapy. Forty-three patients received adjuvant therapy following its introduction into Norwegian national guidelines in 2005. None of the patients received immunotherapy.

**Table 1:** Clinicopathological variables as predictors of disease-specific survival in all 536 NSCLC patients and in SCC and ADC histological subgroups (univariate analyses; log-rank test, unadjusted Cox proportional hazard ratios) [30]

	All patients					Squamous cell carcinoma					Adenocarcinoma				
	*N*(%)	5 year	Median	HR(95% CI)	*P*	*N*(%)	5 year	Median	HR(95% CI)	*P*	*N*(%)	5 year	Median	HR(95% CI)	*P*
Age					0.711					0.654					0.505
≤65	227(42)	57	127	1		106(37)	64	235	1		102(51)	48	54	1	
>65	309(58)	58	NA	0.95 (0.73–1.24)		183(63)	66	NA	0.91(0.61–1.36)		99(49)	49	57	0.87(0.59–1.3)	
Sex					**0.026**					0.108					**0.050**
Female	170(32)	63	190	1		73(25)	73	NA	1		83(41)	56	190	1	
Male	366(68)	55	88	1.4(1.06–1.84)		216(75)	63	235	1.49(0.96–2.31)		118(59)	43	51	1.5(1.01–2.23)	
ECOG perf. status					**0.015**					0.158					**0.003**
0	310(58)	62	235	1		158(55)	69	235	1		122(61)	56	NA	1	
1	190(35)	52	71	1.45(1.09–1.93)		110(38)	61	114	1.47(0.97–2.23)		67(33)	40	50	1.57(1.02–2.4)	
2	36(7)	48	36	1.61(0.83–3.09)		21(7)	67	NA	1.08(0.45–2.6)		12(6)	17	25	3.25(0.96–11.03)	
Smoking					**0.039**					0.19					0.68
Never	17(3)	44	20	1		7(2)	50	19	1		9(5)	44	21	1	
Previous	342(64)	62	235	0.56(0.25–1.24)		182(63)	69	235	0.58(0.14–2.37)		125(62)	50	68	0.69(0.26–1.84)	
Present	177(33)	51	71	0.75(0.33–1.7)		100(35)	60	114	0.82(0.2–3.41)		67(33)	45	57	0.73(0.27–1.99)	
Weight loss					0.961					0.689					0.536
<10%	441(82)	58	127	1		257(89)	66	235	1		184(92)	49	57	1	
≥10%	44(8)	59	NA	0.99(0.63–1.56)		32(11)	62	NA	1.14(0.57–2.28)		17(8)	40	47	1.24(0.59–2.63)	
Surgical procedure					**<0.001**					**<0.001**					<**0.001**
Wedge/Lobectomy	394(74)	63	190	1		197(68)	72	235	1		161(80)	54	104	1	
Pulmonectomy	142(26)	42	30	1.98(1.43–2.74)		92(32)	50	35	1.99(1.28–3.09)		40(20)	25	24	2.66(1.46–4.84)	
Margins					0.129					0.252					**0.018**
Free	489(91)	59	190	1		257(89)	67	235	1		189(94)	50	68	1	
Not free	47(9)	47	57	1.39(0.85–2.29)		32(11)	57	114	1.39(0.73–2.63)		12(6)	0	35	2.33(0.81–6.69)	
pTstage					**<0.001**					**<0.001**					**<0.001**
1	168(31)	72	235	1		83(29)	78	235	1		74(37)	67	190	1	
2	265(49)	57	91	1.74(1.3–2.32)		147(51)	66	NA	1.88(1.22–2.89)		94(47)	43	47	1.94(1.27–2.95)	
3	97(18)	36	30	2.84(1.87–4.31)		56(19)	46	33	2.93(1.62–5.31)		31(15)	16	25	3.48(1.76–6.9)	
4	6(0)	20	15	4.89(0.89–26.9)		3(1)	0	10	17.41(0.22–1371.77)		2(1)	50	13	1.76(0.23–13.27)	
pNstage					**<0.001**					**<0.001**					**<0.001**
0	364(68)	69	235	1		198(69)	77	235	1		133(66)	60	190	1	
1	118(22)	36	35	2.76(1.93–3.94)		73(25)	45	35	3.26(1.99–5.35)		39(19)	25	30	2.41(1.38–4.2)	
2	54(10)	21	19	4.23(2.43–7.37)		18(6)	18	13	7.12(2.44–20.77)		29(15)	23	24	2.88(1.42–5.82)	
Pathological stage					**<0.001**					**<0.001**					**<0.001**
I	256(48)	72	235	1		127(44)	82	235	1		105(52)	65	190	1	
II	194(36)	53	84	1.89(1.42–2.51)		126(44)	60	114	2.5(1.66–3.77)		56(28)	34	43	2.07(1.3–3.28)	
IIIA	86(16)	20	17	4.58(2.87–7.32)		36(12)	23	15	7.15(3.23–15.84)		40(20)	16	24	3.37(1.8–6.33)	
Histology					**0.040**										
SCC	289(54)	65	235	1											
ADC	201(37)	48	57	1.43(1.08–1.89)											
LCC	46(9)	50	83	1.29(0.8–2.08)											
Differentiation					**<0.001**					**0.033**					**0.006**
Poor	231(43)	49	51	1		104(36)	57	84	1		81(40)	38	43	1	
Moderate	240(45)	63	190	0.67(0.5–0.89)		155(54)	70	235	0.63(0.41–0.97)		85(42)	50	68	0.69(0.44–1.07)	
Well	65(12)	70	NA	0.44(0.29–0.66)		30(10)	72	NA	0.47(0.24–0.94)		35(18)	69	NA	0.36(0.21–0.63)	
Vascular infiltration					**<0.001**					**0.029**					**0.012**
No	437(82)	62	235	1		231(80)	69	235	1		172(86)	52	71	1	
Yes	97(18)	38	35	1.89(1.29–2.78)		58(20)	53	71	1.65(0.97–2.82)		27(13)	26	27	1.9(1–3.62)	
Missing	2(0)										2(1)				

Bold numbers are significant

*ADC* adenocarcinoma, *ECOG perf. status* Eastern Cooperative Oncology Group performance status, *HR* hazard ratio, *LCC* large cell carcinoma, *N* number, *pNstage* pathological nodal stage, *pTstage* pathological tumor stage, *SCC* squamous cell carcinoma

### Expression of CTLA-4 in primary tumors and resected metastatic lymph nodes

CTLA-4 staining was predominantly cytoplasmatic and rarely membranous. Staining intensity for CTLA-4 in tumor epithelial cells (T-CTLA-4) was relatively homogenous within each tumor, with variable intensity between tumors. Similarly, staining intensity for CTLA-4 in the different cell types in the stromal compartment (S-CTLA-4) was relatively homogenous within each tumor, and CTLA-4+ cells were dominated by cells morphologically consistent with immune cells. Thus, the stromal CTLA-4 (S-CTLA-4) intensity score is expected to mirror immune infiltration. The stromal component of lymph node metastasis was scarce and difficult to discern from normal lymph node tissue; therefore, it was not scored.

The expression of tumor epithelial and stromal CTLA-4 is presented in Table 2. The percentage of patients with high S-CTLA-4 (50%) was higher than that of T-CTLA-4 (43%) (*P* < 0.001) (percentage of non-missing cores). In PTs, the S-CTLA-4 and T-CTLA-4 mean scores were significantly correlated (*r* = 0.329, *P* < 0.001). The percentages of high T-CTLA-4 in PTs and LN+ (37%) were not significantly different (*P* = 0.547). T-CTLA-4 expression in PTs and LN+ were not significantly correlated; in 56% of cases, T-CTLA-4 scores were concordantly high or low in PTs and LN+, while in 23% of cases, the PT score was high and LN+ score low, and in 21%, the opposite.

**Table 2:** The prognostic impact of tumor epithelial CTLA-4 (T-CTLA-4) and stromal CTLA-4 (S-CTLA-4) in primary tumors and T-CTLA-4 in metastatic lymph nodes on disease-specific survival (DSS) in all patients, and stratified by histology (univariate analyses; log-rank test, unadjusted Cox proportional hazard ratios)

	All patients						Squamous cell carcinoma					Adenocarcinoma				
	*N*(%)	5 year	Median	HR(95% CI)	*P*		*N*(%)	5 year	Median	HR(95% CI)	*P*	*N*(%)	5 year	Median	HR(95% CI)	*P*
Primary tumor																
S-CTLA-4					0.078						**0.013**					0.532
Low	246(46)	55	105	1.00		128(45)		57	114	1.00		96(48)	54	77	1.00	
High	244(46)	59	235	0.78(0.60–1.03)		136(47)		72	235	0.60(0.39–0.90)		89(44)	43	52	1.14(0.75–1.73)	
Missing	45(8)					23(8)						16(8)				
T-CTLA-4					0.121						0.392					**0.037**
Low	278(52)	54	88	1.00		160(55)		64	127	1.00		97(48)	42	47	1.00	
High	213(40)	62	190	0.80(0.60–1.06)		107(37)		68	235	0.83(0.54–1.27)		85(42)	57	104	0.64(0.42–0.98)	
Missing	45(8)					22(8)						19(10)				
Metastatic lymph nodes																
T-CTLA-4					**0.037**						0.114					0.715
Low	76(54)	41	37	1.00		43(57)		54	71	1.00		29(50)	17	25	1.00	
High	44(31)	19	19	1.65(1.03–2.65)		18(24)		23	16	1.79(0.86–3.74)		22(38)	18	21	1.13(0.58–2.21)	
Missing	22(15)					14(19)						7(12)				

Bold numbers are significant results. 5-year survival (%). Median survival (months)

*HR* hazard ratio, *N* number, *S* stroma, *T* tumor

### Associations with clinicopathological variables and immunological markers

There were no significant associations between expression of CTLA-4 in PTs or LN+ and age, sex, Eastern Cooperative Oncology Group (ECOG) performance status, smoking, T-status, N-status, pathological stage, histological subgroup or vascular infiltration. In LN+ patients, high T-CTLA-4 was associated with poorly differentiated tumors (*P* = 0.034).

The S-CTLA-4 mean score in the PTs was, in both stromal and tumor epithelial compartments, extensively correlated with other immunological markers previously analyzed by our group (CD3, CD4, CD8, CD45RO, CD20, PD-1, PD-L1), while T-CTLA-4 in PTs was not (supplementary Table 2) [27, 30–33]. There was a strong and highly significant association between mean LN+ T-CTLA-4 and LN+ T-PD-L1 score (*r* = 0.404, *P* < 0.001*)*, while there were no correlations with the stromal and tumor epithelial PT counterparts.

### Interobserver reliability

Between-scorer agreement was excellent: the intraclass correlation coefficients were 0.894, 0.917 and 0.882, and Kappa values were 0.586, 0.696 and 0.589 for S-CTLA-4, PT T-CTLA-4 and LN+ T-CTLA-4, respectively (all variables, *P* < 0.001).

### Univariate survival analyses

**Fig. 2:**
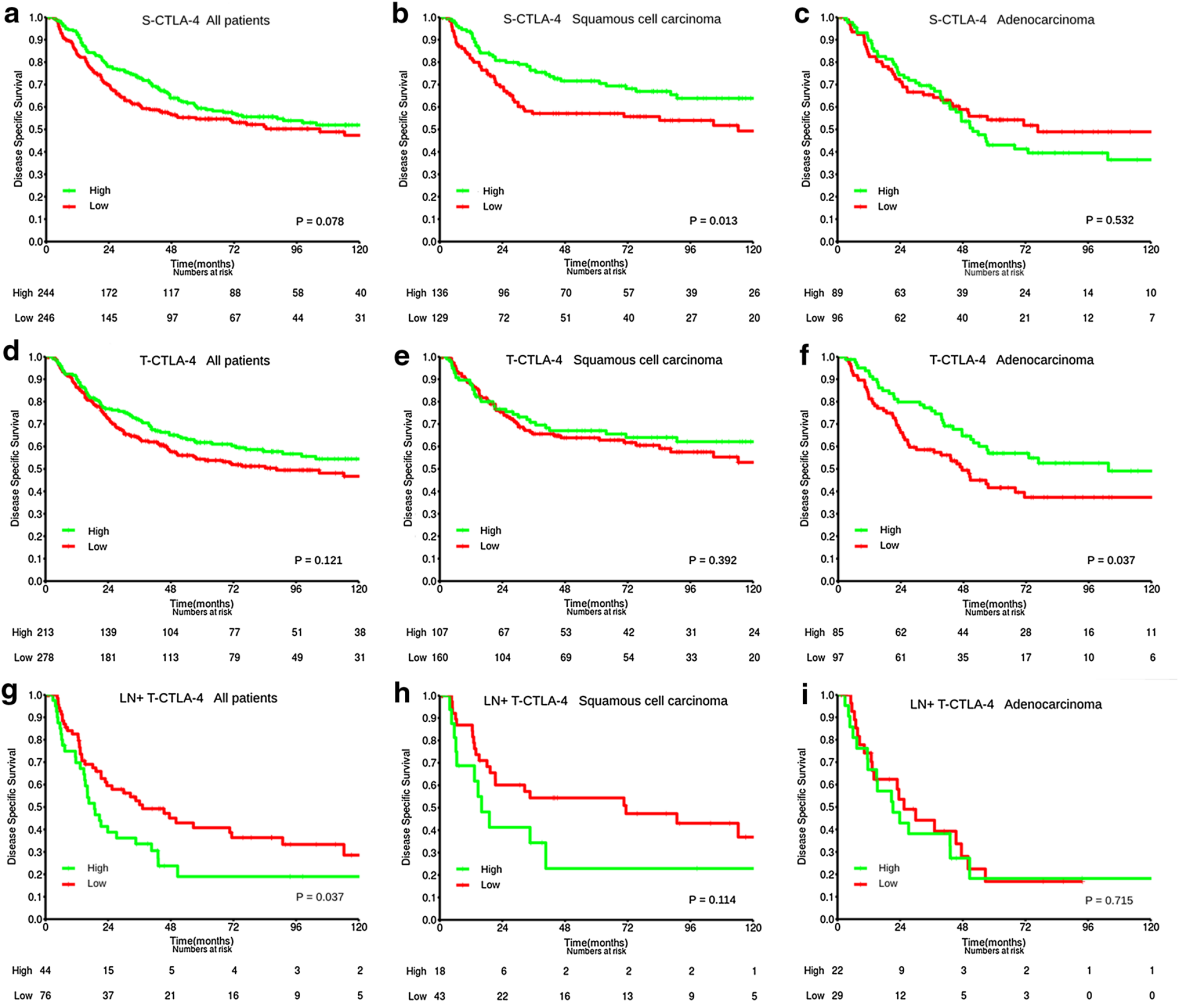
Survival curves. Disease-specific survival curves are shown according to PT expression of S-CTLA-4 (**a**–**c**) and T-CTLA-4 (**d**–**f**) in all patients (**a**, **d**), squamous cell carcinoma (SCC) (**b**, **e**) and adenocarcinoma (ADC) (**c**, **f**) subgroups, and to the expression of T-CTLA-4 in LN+ of all patients with LN+ available (**g**), SCC (**h**) and ADC (**i**) subgroup

The prognostic impact of tumor epithelial and stromal CTLA-4 expression on DSS is presented in Table 2 and Fig. 2 (univariate analyses). Neither tumor epithelial nor stromal CTLA-4 expression predicted DSS for all patients, or for pathological stage subgroups (data not shown). Histological subgroup analyses showed that while high S-CTLA-4 was a positive prognostic factor for DSS (HR 0.60, 95% CI 0.39–0.90, *P* = 0.013) in the SCC subgroup, no association with survival was found in the ADC and large cell carcinoma subgroups. T-CTLA-4 was a positive prognostic factor for DSS only in the ADC group (HR 0.64, 95% CI 0.42–0.98, *P* = 0.037).

By combining the scores of CTLA-4 with other immunological markers previously analyzed by our group (CD3, CD4, CD8, CD45RO, CD20, PD-1, PD-L1), we investigated whether a combination of markers would improve the prognostic impact compared to single marker. Combining PT T-CTLA-4 or S-CTLA-4 scores with each of these markers did not contribute markedly to minimize *P* values or increase stratification according to 5-year DSS, for all patients or in histological subgroups (data not shown).

In metastatic lymph nodes, high expression of CTLA-4 in tumor epithelial cells was associated with an adverse DSS (HR 1.65 95% CI 1.03–2.65, *P* = 0.037).

### Multivariate analysis

Results from the multivariate Cox regression analyses are presented in Table 3. In PTs, S-CTLA-4 was an independent positive prognostic factor for DSS in the SCC subgroup (HR 0.62, 95% CI 0.41–0.93, *P* = 0.021), but not for ADC or all patients. T-CTLA-4 expression in PTs did not have a significant independent prognostic impact in all patients or in histological subgroups. High T-CTLA-4 expression in metastatic lymph nodes was an independent negative predictor of DSS (HR 1.65, 95% CI 1.03–2.65, *P* = 0.039).

**Table 3:** Results of Cox regression analysis summarizing significant independent prognostic factors for disease-specific survival in primary tumors and metastatic lymph nodes (LN+)

	Primary tumors						LN+	
	All patients		Squamous cell carcinoma		Adenocarcinoma		All patients	
	HR(95%CI)	*P*	HR(95%CI)	*P*	HR(95%CI)	*P*	HR(95%CI)	*P*
A. Clinicopathological variables^a^								
Pathological stage		**<0.001** ^b^		**<0.001** ^b^		**<0.001** ^b^		**0.047** ^**b**^
IA	1.00		1.00		1.00			
IB	1.23(0.78–1.96)	0.377	0.98(0.43–2.22)	0.961	1.88(0.99–3.56)	0.052		
IIA	1.68(1.07–2.63)	**0.023**	1.91(0.98–3.74)	0.059	2.88(1.50–5.52)	**0.001**	1.00	
IIB	2.63(1.64–4.12)	**<0.001**	3.35(1.70–6.61)	**<0.001**	3.20(1.50–6.82)	**0.003**	1.09(0.50–2.38)	
IIIA	4.61(2.94–7.23)	**<0.001**	6.86(3.43–13.73)	**<0.001**	4.87(2.55–9.28)	**<0.001**	1.73(1.10–2.71)	
Histology		**0.003** ^b^						NE
Squamous cell carcinoma	1.00							
Adenocarcinoma	1.62(1.20–2.18)	**0.002**						
LCC	0.98(0.59–1.62)	0.924						
Vascular infiltration								NE
No versus yes	1.75(1.26–2.44)	**0.001**	1.56(0.98–2.50)	0.061	1.56(0.91–2.70)	0.108		
Differentiation		**0.005** ^**b**^		0.224		**0.044**		NE
Well	1.00		1.00		1.00			
Moderate	1.80(1.05–3.09)	**0.034**	1.32(0.59–2.92)	0.498	2.24(1.10–4.52)	**0.024**		
Poor	2.36(1.37–4.05)	**0.002**	1.78(0.79–2.03)	0.165	2.39(1.19–4.77)	**0.014**		
Sex								
Female versus male	1.71(1.26–2.33)	**0.001**		NE	1.55(1.00–2.41)	**0.049**		NE
ECOG perf. status		**0.009** ^b^		NE		**0.006** ^b^		NE
0	1.00				1.00			
1	1.49(1.21–1.97)	**0.006**			1.58(1.03–2.43)	**0.037**		
2	1.78(1.00–3.19)	0.051			3.31(1.46–7.47)	**0.004**		
Smoking		**0.017** ^b^		NE		NE		NE
Never	1.00							
Present	0.37(0.19–0.74)	**0.005**						
Former	0.42(0.21–0.84)	**0.014**						
Margins								
Free versus not free		NE		NE	1.41(0.66–3.01)	0.375		NE
B. Immunological markers^c^								
PT S-CTLA-4		NE		**0.021**		NE		
Low versus high			0.62(0.41–0.93)					
PT T-CTLA-4		NE		NE		0.834		
Low versus high					0.95(0.60–1.51)			
LN+ T-CTLA-4								**0.039**
Low versus high							1.65(1.03–2.65)	

Bold numbers are significant results

^a^ In the same model. ^b^ Overall significance as a prognostic factor. ^c^ In separate models. All clinicopathological covariates significant in multivariate analysis (A) are included in each model

*ECOG perf. status* Eastern Cooperative Oncology Group performance status. *HR* hazard ratio. *LCC* large cell carcinoma. *LN+* metastatic lymph nodes. *NE* not entered. *PT* primary tumor

## Discussion

In our large, unselected NSCLC patient cohort, we demonstrate that high expression of CTLA-4 on tumor epithelial cells in regional LN+ independently predicts poor DSS. In contrast, the expression of CTLA-4 in PTs was not significantly associated with outcome in all patients. However, a high stromal CTLA-4 expression independently predicted prolonged DSS for patients with SCC histology. In addition, we observed no correlation between CTLA-4 expression in the PTs and the LN+. Strikingly, this illustrates that phenotypical differences between the tumor microenvironments of PTs and LN+ may result in diverging impacts on NSCLC prognosis.

To our knowledge, this is the largest published study analyzing prevalence and prognostic importance of CTLA-4 expression in NSCLC, and the first to assess both tumor and stromal cells of NSCLC PTs as well as matched LN+ [23–26, 34–36]. The antibody used was subject to careful validation, and between-scorer agreement was excellent.

With our methodology, we were not able to score stromal CTLA-4 expression in LN+. In light of the independent impact of S-CTLA-4 in PTs, the delineation of stroma surrounding tumor islets in LN+ needs to be clarified, allowing assessment of CTLA-4 in the tumor microenvironment of N+ disease in future studies. Due to their intimate interaction with tumor cells, the assessment of CTLA-4+ immune cells infiltrating the tumor epithelial compartment may have added significant value to the study. However, a confirmatory methodology, such as immunofluorescence dual staining of CD45 and CTLA-4, would be required to achieve a precise evaluation of these cells. Moreover, CTLA-4 expression in cores from central tumor and invasive margin may have differing prognostic value, but consistent sampling from distinct tumor areas within the tumor epithelial compartment were not included in the TMAs in this study [7]. Potential heterogeneity in CTLA-4 expression within the tumor tissue was minimized by analyzing cores from two to four areas of tumor and of stroma. Manual, semi-quantitative scoring is a time- and cost-efficient method, but the interpretation of staining intensity is to some degree subjective and for future validation purposes, whole tissue slides and digital automated scoring of immunohistochemistry should be considered.

The positive prognostic value of CTLA-4 expression in PTs was limited to histological subgroups, and was not a significant predictor of outcome in all pathological stages. Thus, CTLA-4 is not considered a good candidate for a NSCLC TNM-Immunoscore.

Our most striking finding was the independent negative prognostic impact of high T-CTLA-4 expression in metastatic lymph nodes. T-CTLA-4 in LN+ was not significantly correlated to the expression of T-CTLA-4 in PTs. This demonstrates heterogeneity of tumor cell CTLA-4 expression between PTs and LN+, probably brought about by genetic and epigenetic alterations acquired during tumor progression and metastasis [37]. In NSCLC, evidence supports that tumor cells sampled from the PT and metastatic sites display molecularly distinct characteristics; genetic heterogeneity between tumor cells in PTs and LN+ with regard to EGFR (epidermal growth factor receptor) and KRAS (Kirsten ras oncogene homolog) status and mutational profiles of other actionable genes is not infrequent [38, 39]. Additionally, the observed heterogeneity of T-CTLA-4 expression within PTs in our study, combined with a lack of correlation with other immune markers, supports that CTLA-4 upregulation in tumor cells is mainly induced by intrinsic oncogenic mechanisms, rather than regulated by the surrounding inflammatory microenvironment, as was previously reported for gastric cancer [40].

In line with our results in patients with LN+, recent studies in other carcinomas have reported that tumor CTLA-4 expression is a negative prognostic factor. This may indicate that increased CTLA-4 expression is associated with immunosuppression and acceleration of disease progression and metastasis, possibly mediated by tumor cell synthesis of soluble CTLA-4 [41–43]. On the other hand, a lack of association with outcome, as well as a favorable prognostic impact of T-CTLA-4, has also been reported [40, 44]. A study demonstrating in vitro induction of tumor cell apoptosis upon CTLA-4 engagement with B7 ligands supports a potential positive prognostic impact of T-CTLA-4 [45]. Few studies have investigated the prognostic impact of CTLA-4 expression in NSCLC. Two large studies reported high gene expression of *CTLA*-*4* to mediate a negative impact, and no significant association with survival, respectively [23, 25]. Similar to a smaller study by Salvi et al., we observed no significant prognostic impact of high PT T-CTLA-4, when assessing protein expression by IHC and applying the 14D3 mAb, except for a positive association with DSS for ADC patients, which was not significant in multivariate analysis [24]. Apparently, these studies illustrate a dual role of CTLA-4 expression in tumor cells, and highlight the importance of further investigating mechanisms of upregulation, impact on prognosis and differences in CTLA-4 expression in tumor cells by cancer subtypes.

High stromal CTLA-4 expression was associated with an independent positive outcome for the SCC subgroup, and showed a positive trend for all patients. Even though several stromal cell types were positive for CTLA-4 in the NSCLC tumors, immune cell staining intensity was the predominant determinant of the S-CTLA-4 score. A positive impact of CTLA-4 on outcome has also been reported for the expression in breast cancer related interstitial lymphocytes [41]. CTLA-4 in T cells is normally upregulated only upon activation. Hence, high stromal CTLA-4 expression presumably reflects a tumor microenvironment highly infiltrated by activated immune cells, even though immunosuppressive subtypes such as regulatory T cells and exhausted cytotoxic T cells also express CTLA-4 [46]. We infer that the positive association between S-CTLA-4 and survival in SCC tumors can be explained by the presence of a tumor microenvironment in which anti-tumor immunity properties dominate. The contrasting lack of prognostic effect for S-CTLA-4 in ADC patients may illustrate existing differences in the balance between activated immune cells with immunosuppressive and anti-tumor immune properties between histological NSCLC subgroups. This is in line with the differences observed in immunotherapy treatment efficacy according to histological subgroups in NSCLC [22, 47, 48]. Furthermore, we observed S-CTLA-4 to be extensively correlated with other immune markers, especially with S-CD8 and S-PD-L1, which were recently found to be independent positive prognostic markers in the same patient population [27, 30]. Interestingly, it has been suggested that a pre-existing immune-active tumor microenvironment is what mediates the anti-tumor activity of CTLA-4 blockade; hence, one may speculate that stromal CTLA-4 expression may have potential as a predictive marker for anti-CTLA-4 treatment in NSCLC patients [49].

In conclusion, we hypothesize that CTLA-4 expression by tumor cells in locoregional LN+, but not PTs, may predict poor survival in NSCLC patients. If validated in larger, confirmatory studies, tumor cell CTLA-4 expression in LN+ is a promising prognostic marker, readily available in surgically treated patients. Its prognostic impact should also be investigated for other metastatic sites. Furthermore, despite its acknowledged immunosuppressive mechanism of action, we hypothesize that high CTLA-4 expression in the stromal compartment mirrors immune cell activation, and speculate that analysis of S-CTLA-4 may allow tailored checkpoint blockade to individual patients.

## Electronic supplementary material

Below is the link to the electronic supplementary material.

Supplementary material 1 (PDF 1106 kb)

## Abbreviations

ADC: Adenocarcinoma
APC: Antigen-presenting cell
CTLA-4: Cytotoxic T-lymphocyte-associated antigen-4
DSS: Disease-specific survival
ECOG: Eastern Cooperative Oncology Group
EGFR: Epidermal growth factor receptor
KRAS: Kirsten ras oncogene homolog
LCC: Large cell carcinoma
LN+: Metastatic lymph nodes
N+: Node positive
NSCLC: Non-small cell lung cancer
PD-1: Programmed death 1 receptor
PD-L1: Programmed death ligand 1
PT: Primary tumor
S-: Stromal
SCC: Squamous cell carcinoma
T-: Tumor epithelial
TMA: Tissue microarray
TNM: Tumor lymph node metastasis stage
