# Supplementary material for: CTLA-4 expression in the non-small cell lung cancer patient tumor microenvironment: diverging prognostic impact in primary tumors and lymph node metastases
Source: Cancer Immunol Immunother. 2017 Jul 13;66(11):1449–61. doi: 10.1007/s00262-017-2039-2 (PMC5645427; doi:10.1007/s00262-017-2039-2)
Supplement: Supplementary file 1 — Supplementary material 1 (PDF 1106 kb) [file 262_2017_2039_MOESM1_ESM.pdf]

Supplementary Table 1 Clinicopathological variables as predictors of disease-specific survival (DSS) in 142 N+ NSCLC patients (univariate analyses; log-rank test, unadjusted Cox proportional hazard ratios).

	All patients					Squamous cell carcinoma					Adenocarcinoma				
	**N**	**5 year**	**Median**	**HR(95% CI)**	**P**	**N**	**5 year**	**Median**	**HR(95% CI)**	**P**	**N**	**5 year**	**Median**	**HR(95% CI)**	**P**
**Age**					0.883					0.597					0.110
≤65	69	29	24	1.00		35	46	33	1.00		32	5	23	1.00	
>65	73	32	25	0.97(0.64-1.47)		39	39	21	1.18(0.64-2.14)		26	30	43	0.59(0.31-1.13)	
**Sex**					0.370					0.966					0.184
Female	35	27	43	1.00		10	40	21	1.00		21	22	43	1.00	
Male	107	31	21	1.25(0.77-2.05)		64	43	28	0.98(0.41-2.33)		37	14	15	1.55(0.81-2.99)	
**ECOG perf. status**					0.689					0.987					0.589
0	78	30	27	1.00		37	41	21	1.00		35	19	30	1.00	
1	53	33	23	1.06(0.68-1.64)		32	45	33	1.05(0.57-1.94)		18	14	15	1.28(0.66-2.51)	
2	11	25	25	1.45(0.62-3.42)		5	38	25	1.07(0.25-4.60)		5	25	8	1.71(0.50-5.81)	
**Smoking**					0.121					0.287					0.451
Never	5	0	8	1.00		3	0	6	1.00		2	0	8	1.00	
Previous	82	32	35	0.39(0.14-1.12)		41	41	35	0.36(0.08-1.56)		36	14	25	0.39(0.09-1.78)	
Present	55	31	21	0.35(0.13-1.00)		30	45	25	0.33(0.08-1.41)		20	22	24	0.42(0.10-1.82)	
**Weightloss**					0.148					**0.029**					0.646
<10%	126	32	25	1.00		66	44	35	1.00		51	19	24	1.00	
≥10%	16	0	14	1.59(0.84-3.01)		8	29	6	2.78(1.06-7.30)		7	0	15	1.23(0.51-2.94)	
**Surgical procedure**					0.272					0.616					0.053
Wedge/Lobectomy	73	31	41	1.00		31	47	41	1.00		38	20	43	1.00	
Pulmonectomy	69	29	19	1.26(0.83-1.92)		43	39	21	1.17(0.64-2.15)		20	10	12	1.88(0.98-3.60)	
**Margins**					0.415					0.727					0.452
Free	124	32	25	1.00		63	45	28	1.00		52	19	25	1.00	
Not free	18	18	23	1.28(0.71-2.30)		11	31	35	1.15(0.53-2.47)		6	0	8	1.49(0.53-4.20)	
**Tstage**					0.101					**0.047**					0.268
1	28	41	44	1.00		13	*66*	NR	1.00		12	16	43	1.00	
2	77	31	24	1.67(0.92-3.02)		41	39	25	2.42(0.93-6.31)		34	22	24	1.16(0.52-2.59)	
3	33	21	16	1.98(1.00-3.89)		17	36	17	2.20(0.74-6.60)		12	0	10	2.02(0.77-5.26)	
4	4	0	15	3.57(1.02-12.49)		3	0	5	8.80(1.63-47.45)		0				
**Nstage**					**0.024**					**0.013**					0.315
1	97	36	36	1.00		59	50	41	1.00		33	19	30	1.00	
2	45	20	17	1.65(1.06-2.56)		15	14	7	2.82(1.17-2.27)		25	18	21	1.39(0.73-2.67)	
**Pathological stage**					**0.044**					0.093					0.366
IIA	56	42	43	1.00		33	60	84	1.00		21	20	27	1.00	
IIB	14	38	18	1.09(0.50-2.38)		10	40	16	1.56(0.61-4.01)		4	38	47	0.56(0.13-2.43)	
IIIA	72	18	17	1.73(1.10-2.71)		31	23	15	2.03(1.06-3.91)		33	8	21	1.37(0.71-2.64)	
**Histology**					0.421										
SCC	74	42	28	1.00											
ADC	58	17	24	1.32(0.85-2.04)											
LCC	10	19	19	1.37(0.58-3.25)											
**Differentiation**					0.196					0.441					0.476
Poor	79	25	23	1.00		34	38	19	1.00		35	11	25	1.00	
Moderate	55	33	21	0.88(0.57-1.35)		36	44	33	0.87(0.47-1.60)		19	18	15	0.95(0.49-1.84)	
Well	8	57	NR	0.30(0.07-1.23)		4	50	41	0.30(0.04-2.21)		4	75	NR	0.31(0.04-2.28)	
**Vascular infiltration**					0.059					**0.036**					0.783
No	105	35	30	1.00		55	49	41	1.00		45	15	24	1.00	
Yes	36	18	18	1.56(0.98-2.49)		19	20	18	2.01(1.03-3.89)		12	23	27	0.90(0.41-1.96)	

Note: Bold numbers are significant. Abbreviations: Adenocarcinoma. ECOG perf.status, Eastern Cooperative Oncology Group performance status. HR, hazard ratio. LCC, large cell carcinoma. N, number. Nstage, Nodal stage. SCC, squamous cell carcinoma. Tstage, Tumor stage.

**Supplementary Table 2**: Spearman rank correlations between mean scores of CTLA-4 and clinicopathological variables and between compartments			
	**PT S-CTLA-4**	**PT T-CTLA-4**	**LN+ T-CTLA-4**
**S-CTLA-4**		0.391**	ns
**T-CTLA-4**	0.391**		ns
**LN+ T-CTLA-4**	ns	ns	
**S-CD3**	.289**	ns	ns
**T-CD3**	.235**	ns	ns
**LN+ T-CD3**	ns	ns	ns
**S-CD4**	.249**	-.112*	ns
**T-CD4**	.168**	ns	ns
**LN+ T-CD4**	ns	ns	ns
**S-CD8**	.313**	ns	ns
**T-CD8**	.225**	.141*	ns
**LN+ T-CD8**	ns	ns	ns
**S-CD45RO**	.165**	ns	ns
**T-CD45RO**	.147*	ns	ns
**LN+ T-CD45RO**	ns	ns	ns
**S-CD20**	0.302**	ns	ns
**T-CD20**	0.205**	ns	ns
**LN+ T-CD20**	ns	ns	0.231*
**S-PD-1**	.249**	ns	ns
**T-PD-1**	.165**	ns	ns
**LN+ T-PD-1**	ns	ns	ns
**S-PD-L1**	.354**	ns	ns
**T-PD-L1**	.192**	.101 *	ns
**LN+ T-PD-L1**	ns	ns	.404**

Abbreviations: * significant at <0.05. ** significant at <0.001. LN+, metastatic lymph node. ns, not significant (P ≥0.05). PT, primary tumor. S, stroma. T, tumor

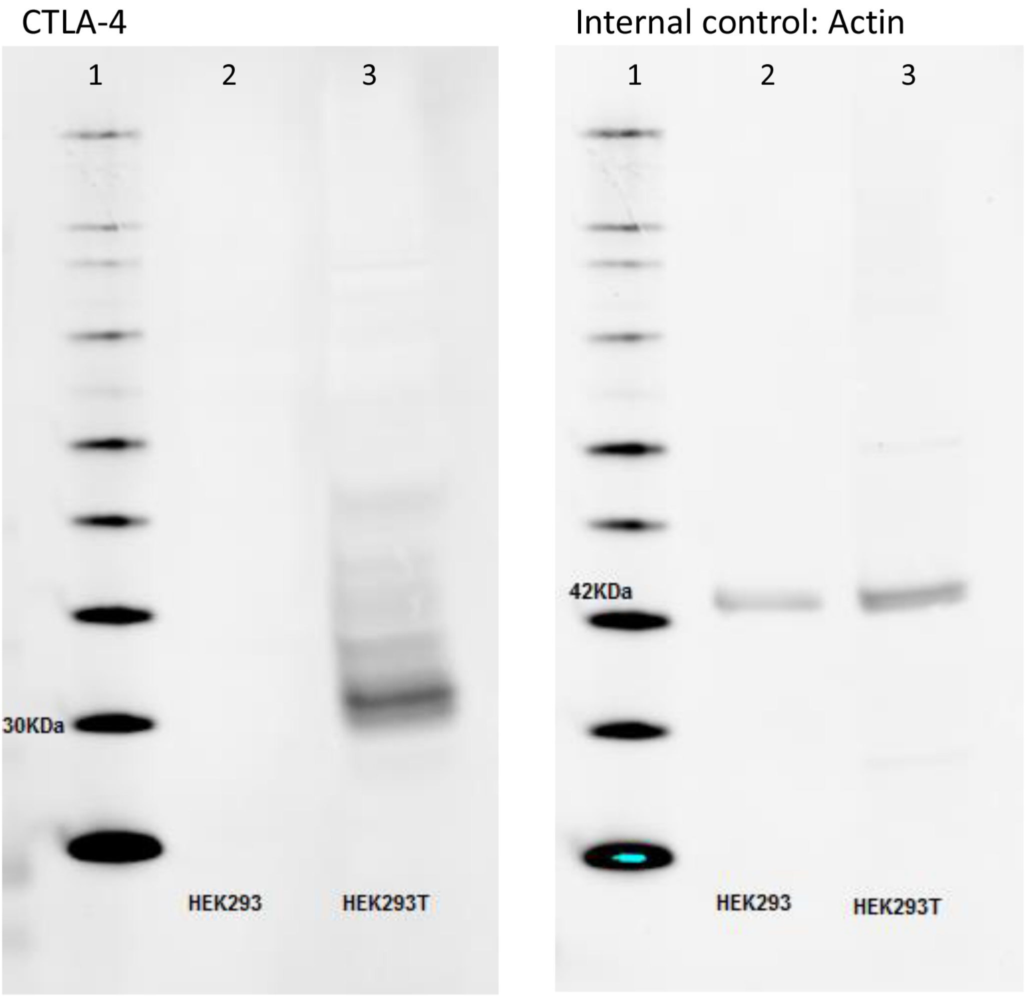

Supplementary Fig.1 Antibody validation

CTLA-4 (CD152, Cat#14-1529, clone 14D3, eBioscience). 1, Molecular weight marker (MagicMark XP); 2, Empty vector (#LY5000001/negative control); 3, Transient overexpressed human HEK293T cell lyastes for CTLA-4 (#LY417438). The most prominent band (30kDa) represents the observed molecular weight of the detected protein, which corresponds with the predicted weight (24.66 kDa) provided by the manufacturer.
